# Glioma cancer stem cells modulating the local tumor immune environment

**DOI:** 10.3389/fnmol.2022.1029657

**Published:** 2022-10-10

**Authors:** Imran Khan, Sadaf Mahfooz, Busra Karacam, Elif Burce Elbasan, Kerime Akdur, Hasiba Karimi, Ayten Sakarcan, Mustafa Aziz Hatiboglu

**Affiliations:** ^1^Department of Molecular Biology, Beykoz Institute of Life Sciences and Biotechnology, Bezmialem Vakif University, Istanbul, Turkey; ^2^Department of Neurosurgery, Bezmialem Vakif University Medical School, Istanbul, Turkey; ^3^Bezmialem Vakif University Medical School, Istanbul, Turkey

**Keywords:** glioma stem cells, glioblastoma, tumor immune response, systemic immune response, tumor microenvironment

## Abstract

Glioma stem cells (GSCs) drive the resistance mechanism in glioma tumors and mediate the suppression of innate and adaptive immune responses. Here we investigate the expression of mesenchymal-epithelial transition factor (c-Met) and Fas receptor in GSCs and their role in potentiating the tumor-mediated immune suppression through modulation of tumor infiltrating lymphocyte (TIL) population. Tumor tissues were collected from 4 patients who underwent surgery for glioblastoma. GSCs were cultured as neurospheres and evaluated for the co-expression of CD133, c-Met and FasL through flow cytometry. TILs were isolated and evaluated for the lymphocyte subset frequencies including CD3 +, CD4 +, CD8 +, regulatory T cells (FOXP3 + CD25) and microglia (CD11b + CD45) using flow cytometry. Our findings revealed that a significant population of GSCs in all four samples expressed c-Met (89–99%) and FasL (73–97%). A significantly low microglia population was found in local immune cells ranging from 3 to 5%. We did not find a statistically significant correlation between expressions of c-Met + GSC and FasL + GSC with local and systemic immune cells. This may be regarded to the small sample size. The percent c-Met + and FasL + GSC population appeared to be related to percent cytotoxic T cells, regulatory T cells and microglia populations in glioblastoma patients. Further investigation is warranted in a larger sample size.

## Graphical Abstract

The schematic representation of the study design. 
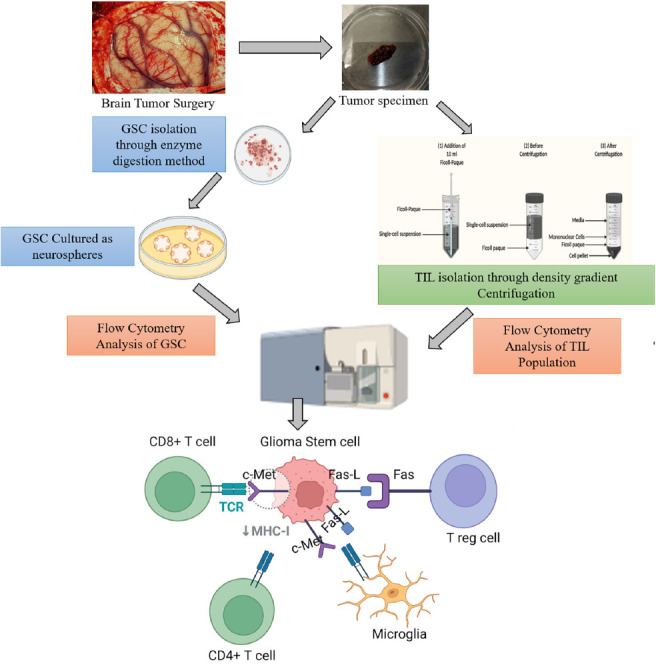

## Introduction

Glioblastomas are thought to be immunologically cold tumors and poorly infiltrated by immune cells (Weenink et al., 2020). Patients with glioblastoma are shown to be immunosuppressed both locally and systemically (Dix et al., 1999; Waziri, 2010). Several mechanisms such as hypoxia, immunosuppressive cytokine production, reduced T-cell proliferation, and effector response, activation of FoxP3 + regulatory T (Treg) cells and myeloid-derived suppressor cells (MDSC) mediate the immunosuppression in glioblastoma (Mirghorbani et al., 2013; Razavi et al., 2016). The current understanding of tumor-mediated immunosuppression in patients with glioblastoma shows that tumor cells suppress lymphocyte proliferation and interleukin-6 (IL-6) production *via* secreting prostaglandin E2 (PGE2) and transforming growth factor-β2 (TGF- β2) (Parney, 2012; Rolle et al., 2012). The majority of tumor-infiltrating immune cells are constituted by microglia, which are central nervous system (CNS) resident macrophages. Glioblastoma tumors recruit immunosuppressive tumor-associated macrophages (TAM) in the tumor microenvironment (Wu et al., 2010). These M2-TAMs play a tumor-promoting role and enhance neovascularization in glioblastoma tumors (Pyonteck et al., 2013).

C-Met is a receptor tyrosine kinase that promotes cell proliferation and migration in gliomas (Abounader and Laterra, 2005). Joo et al. demonstrated that GSCs express high levels of c-Met and substantiate its functional requisite for maintaining the stemness in GSC (Joo et al., 2012). Several studies suggest that c-Met activation drives immune evasion by induction of T-cell tolerance and impairment of dendritic cell functions (Okunishi et al., 2005; Benkhoucha et al., 2010). Thus, inhibition of c-Met enhances the mobilization of reactive neutrophils in the tumor microenvironment and subsequently improves the response to immunotherapy (Glodde et al., 2017). Identifying the factors that facilitate the c-Met-driven immune evasion would help develop more effective immunotherapies against GBM.

The Fas-FasL system is crucial in suppressing inflammation in a normal healthy brain (Taylor, 1996; Neumann, 2000). Fas-FasL-mediated apoptosis maintains T-cell development, selective autoreactive B-cell deletion, and regulates CD8^+^ T cells and NK cells (Rouvler et al., 1993; Russell et al., 1993; Ramsdell et al., 1994; Rathmell et al., 1995; Volpe et al., 2016). Malignant brain tumors, including glioblastoma, neuroblastoma, and medulloblastoma, express Fas and FasL. Interestingly, FasL expressing brain tumor cells can induce apoptosis in tumor-infiltrating immune cells and evade host immune response (Céfai et al., 2001; Didenko et al., 2002).

With this report, we investigated the role of c-Met and FasL in modulating the local and systemic immune system response in patients with glioblastoma. We hypothesized that the expression of c-Met and Fas on GSCs might be correlated with immunosuppressive immune cell subsets in the local and systemic immune systems of patients with glioblastoma.

## Materials and methods

### Patients’ data

Patients who underwent surgery for primary glioblastoma were included in this study. All patients were treated with standard surgical resection and radiation therapy with concurrent temozolomide treatment. Patients with recurrent tumor and those who received previous radiation or chemotherapy were excluded. The patients’ characteristics, including age, sex, Karnofsky performance status (KPS), histology of tumors, and tumor volume, were retrospectively reviewed. Also, matched healthy controls were enrolled to investigate the difference in circulating lymphocyte subset frequencies between healthy controls and glioblastoma patients. All the methodology implemented in the study was as per Helsinki’s world medical association declaration guidelines. The Ethical Committee of Bezmialem Vakif University approved the study (Ethical no. 2019-6/14).

### Glioma stem cells isolation and culture

Resected tumor tissues were transferred to the research laboratory. Tumor tissues (200–500 mg) were minced using a sterile surgical blade and then enzymatically digested using liberaseTM (Roche, South San Francisco, USA) 0.2 unit/ml in 10 ml RPMI-1640 for 30 min at 37°C. Then the tumor tissue was washed with 20 ml RPMI-1640 medium to remove surrounding blood and was homogeneously divided into two pieces for GSCs isolation and tumor-infiltrating lymphocytes (TILs) isolation. After washing, the digested tumor tissues were subjected to RBC lysis to remove the remaining blood cells. Later the tumor tissue digest was strained through a 100 μm strainer followed by a 40 μm strainer. The strained cells were cultured in a neurosphere culture medium composed of Dulbecco’s modified Eagle’s medium/F-12 mediums supplemented with 1% antibiotics, B27 growth factor and 20°ng/ml each of fibroblast growth factor (FGF-2) (Sigma, USA) and epidermal growth factor (EGF) (Sigma, USA). Cultures were maintained at standard growth conditions and observed for neurosphere development. Neurospheres were confirmed for the expression of stem cell markers CD133-FITC, and Nestin-DAPI (BD Biosciences, USA) through immunofluorescence using Cytation 5 (BioTek, USA).

### Peripheral blood mononuclear cells isolation

Peripheral blood samples were collected from patients on the day of surgery before tumor removal and from matched healthy control in heparin blood collection tubes (Greiner Bio-one, Kremsmünster, Austria). PBMCs were isolated using Histopaque density gradient centrifugation at 2,100 rpm for 25 min. Isolated PBMCs were resuspended in phosphate buffer saline (PBS) and prepared for flow-cytometry analysis to determine the subpopulation of immune cells.

### Flow cytometry for glioma stem cells

The samples were analyzed using flow cytometry (S3e™ Cell Sorter BioRad, Germany). Neurospheres were washed and blocked with 3% Bovine Serum Albumin for 1 h. Then the cells were fixed with 4% paraformaldehyde and permeabilized with Tween 20 (0.05%). Finally, the stem cells were co-stained with CD133-FITC, Nestin-DAPI (BD Biosciences, USA), FasL (Biolegend, USA) and c-Met (Thermo Fischer Scientific, USA), and incubated in the dark for 2 h at room temperature. Later, the samples were incubated for 2 h at room temperature with a fluorochrome-tagged secondary antibody.

### Tumor infiltrating lymphocyte isolation

Tumor infiltrating lymphocyte (TIL) isolation from tumor tissues was performed using Ficoll-Paque density-based separation as previously described (Tan and Lei, 2019). Tumor tissues were mechanically minced using a sterile surgical blade and strained through a 70 μm cell strainer. Later 10°ml RPMI-1640 medium was added cells pellet, followed by carefully adding 3°ml Ficoll-Paque medium to the bottom of the same tube using a serological pipette. This density gradient suspension was centrifuged at 1,250 g for 20 min at 20°C. The white layer of mononuclear cells formed at the intersection is carefully removed and resuspended in phosphate buffer saline (PBS) and prepared for flow-cytometry analysis to determine the subpopulation of immune cells.

### Immunophenotyping of immune cells

Immune subset frequencies in PBMCs and TILs samples were evaluated by flow cytometry. The PBMCs and TILs were stained with fluorochrome-tagged antibodies from Biolegend. All samples were blocked with 3% bovine serum albumin for 30 min at room temperature. Antibodies were added as per manufacturer’s recommendation for T lymphocytes (CD3^+^), cytotoxic T lymphocytes (CD3^+^ CD8^+^), T helper lymphocytes (CD3^+^ CD4^+^), T regulatory (Treg) (CD25 + FOXP3) and microglia (CD11b + CD45) and incubated overnight at 4°C in dark. Later, Fas (Biolegend, San Diego, USA), FasL (Biolegend, San Diego, USA), and c-Met (Thermo Fischer Scientific, USA) antibodies were added and samples were incubated for 2 h. To detect T reg cells, samples were fixed and permeabilized using FOXP3 Fix/Perm Buffer (Biolegend, USA) and the samples were incubated at 4°C overnight in the dark. All the samples were analyzed using flow cytometry.

## Results

### Glioma stem cells expressed high levels of c-Met and FasL

**FIGURE 1:**
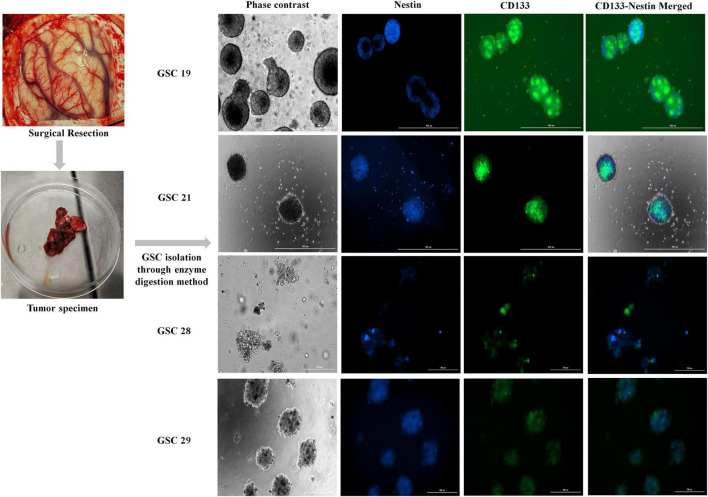
Fluorescent images of *ex vivo* neurosphere cultures of glioma stem cells isolated from tumor specimens; GSC19, GSC21, GSC28, and GSC29 co-stained for stem cell markers Nestin and CD133.

Four patients who underwent surgical resection for glioblastoma were reviewed. Patients’ characteristics were presented in Table 1. Four patient tumor-derived neurospheres were generated and tested for the expression of stem cell markers CD133 and Nestin (Figure 1). The single-cell suspension of neurospheres were analyzed for the dual expression of CD133 with c-Met and FasL through flow cytometry. The average percentage of c-Met was 95.9% (range 89–99%) and FasL 88.9% (range 97.8–73.6%) in CD133 positive GSCs.

**TABLE 1:** Characteristics of patients who underwent surgical resection for glioblastoma.

Characteristic	Patient 1	Patient 2	Patient 3	Patient 4
Age, years	59	70	65	66
Gender	Male	Male	Male	Female
Pathology	Glioblastoma	Glioblastoma	Glioblastoma	Glioblastoma
IDH status	Wild	Wild	Wild	Wild
Location	Occipital	Parietal	Temporal	Occipital
Tumor volume (cm^3^)	137.7	82.6	41.8	109.3
Radiotherapy	Yes	No	Yes	Yes
Chemotherapy	Yes	No	Yes	Yes
PFST (months)	9	n/a	17.5	4
Overall survival time (months)	13.5	1*	18.5	18

*Patient 2 deceased due to pulmonary embolism, a non-tumoral reason. IDH, Isocitrate dehydrogenase; PFST, Progression free survival time; n/a, Not applicable.

### c-Met and FasL expression on glioma stem cells modulate the local immune response in tumor microenvironment

**FIGURE 2:**
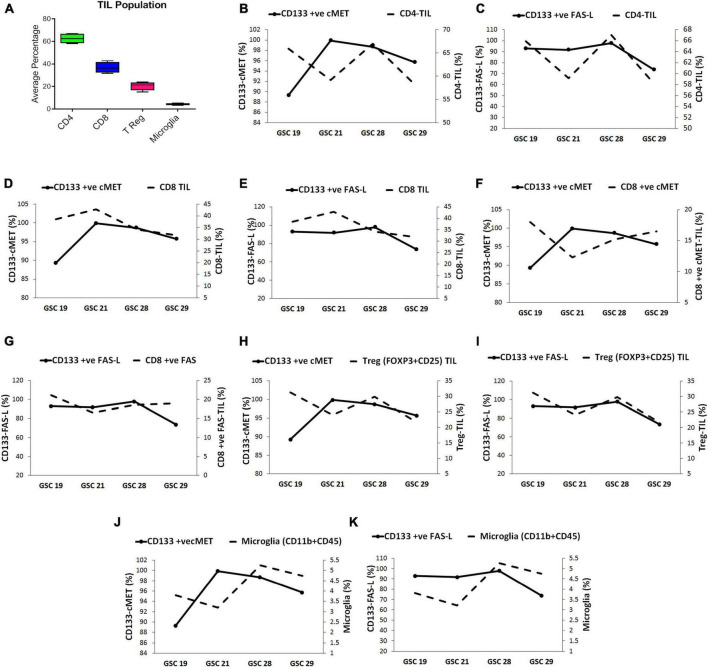
Glioma stem cells modulate the tumor infiltrating lymphocyte (TIL) population in glioblastoma tumors. **(A)** The average percentage of immune cell subsets in TILs. The correlation between GSC expressing c-Met and FasL with TIL subset population was evaluated through multivariable plots; **(B)** CD133^+^ c-Met-GSCs were inversely related to CD4^+^ -TIL **(C)** CD133^+^ FasL-GSCs were directly related to CD4^+^ -TIL. **(D)** CD133^+^ c-Met-GSCs were directly related to CD8^+^ -TIL. **(E)** CD133^+^ FasL-GSCs were inversely related to CD8^+^ -TIL. **(F)** CD133^+^ c-Met-GSCs were inversely related with CD8^+^ ve c-Met-TILs. **(G)** CD133^+^ FasL-GSCs were directly related to CD8^+^ ve Fas-TILs. **(H)** CD133^+^ c-Met-GSCs were inversely related to T reg-TIL **(I)** CD133^+^ FAS-L-GSCs were directly related to T reg-TIL. **(J)** CD133^+^ c-Met-GSCs were inversely related to Microglia **(K)** CD133^+^ FasL-GSCs were directly related to Microglia.

Tumor infiltrating lymphocyte (TILs) obtained from glioblastoma patient’s tumor specimens (*n* = 4) were evaluated for the presence of CD4^+^, CD8^+^, T reg (CD25+ FOXP3), and microglia (CD11b + CD45), through flow cytometry. As depicted in Figure 2A, the highest average percent population 62.56% (range 67–58.15%) was of CD4^+^ cells, followed by, 36.82% (range 42.85–31.72%) of CD8^+^, 20.50% (range 24.1–15.22%) of T reg cells and 4.25% (range 5.26–3.2%) of microglia.

Since the sample size was small to reach a statistically significant conclusion, the correlation between GSCs expressing c-Met and FasL (CD133 + c-Met and CD133 + FasL) with TILs was assessed by comparing trends in multivariable plots. The CD4^+^ TIL population presented an inverse relation with the CD133^+^ c-Met GSCs except for GSC 29 sample. On the contrary, they were directly related to the CD133^+^ FasL GSCs (Figures 2B,C). The sample size was small to reach a statistically significant conclusion.

Collectively comparing the trend in all samples, the highest percentage of CD133^+^ c-Met GSCs and CD8^+^ TIL were found in GSC21. Moreover, in samples GSC19, GSC28, and GSC29, the number of CD133^+^ c-Met and CD8^+^ TIL decreased consistently. This trend indicates CD8^+^ TIL population seemed to be directly related to the CD133^+^ c-Met GSCs (Figure 2D. On the other hand, CD133^+^ FasL GSCs were high and CD8^+^ TIL was low in the GSC21 sample, a similar pattern was observed in all samples. This indicates an inverse relation between CD133^+^ FasL GSCs and CD8^+^ TIL (Figure 2E. However, this trend was opposite for CD8^+^ TIL expressing c-Met and Fas (CD8^+^ ve c-Met TIL and CD8^+^ ve Fas TIL) compared with GSCs CD133^+^ c-Met and CD133^+^ FasL GSCs populations (Figures 2F,G). The CD8^+^ ve c-Met TIL frequencies were inversely related with GSCs, CD133^+^ c-Met. On the other hand, CD8^+^ ve Fas TIL frequencies were directly related to CD133^+^ FasL GSCs populations.

Treg TIL population seems to be inversely related to the CD133^+^ c-Met GSCs population except for GSC 29 sample; however, they were directly related to the CD133^+^ FasL GSCs population (Figures 2H,I). The microglia (CD11b + CD45) population seemed inversely related to CD133^+^ c-Met GSCs except for sample GSC 21. On the other hand, they were directly related to the CD133^+^ FasL GSCs population (Figures 2J,K).

### c-Met and FasL expression on glioma stem cells modulate peripheral circulating immune cells

**FIGURE 3:**
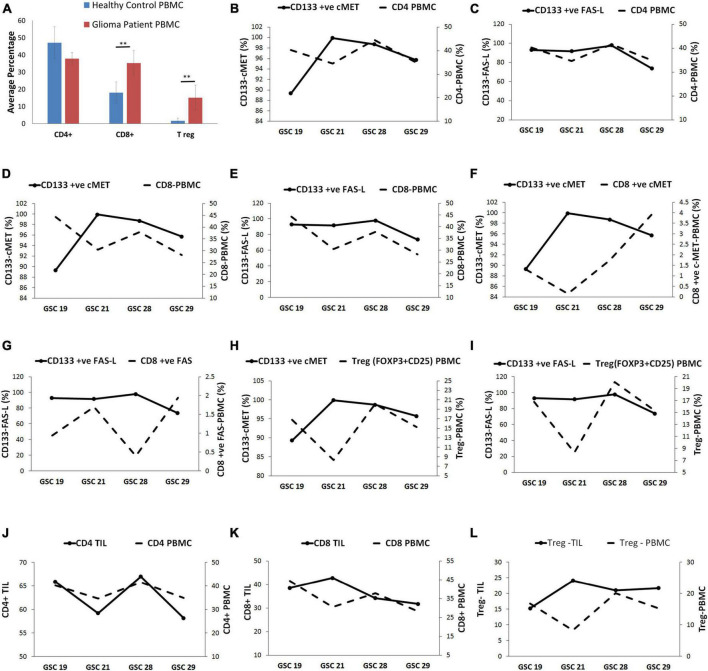
Glioma stem cells were linked with a systemic immunosuppressed phenotype of glioblastoma patients. **(A)** The immune cell frequency distribution in glioma patients and healthy controls. **(B)** The CD4^+^ PBMCs displayed inverse relation with CD133^+^ c-Met-GSCs except for GSC 29 sample. **(C)** CD4^+^ PBMCs were directly related to CD133^+^ FasL-GSCs. **(D)** The CD8^+^ PBMCs were inversely related to CD133^+^ c-Met GSCs except for GSC 29. **(E)** The CD8^+^ PBMCs are directly related to CD133^+^ FasL-GSCs. **(F)** CD8^+^ ve c-Met-PBMC were inversely related to CD133^+^ c-Met-GSCs. **(G)** CD8^+^ ve Fas-PBMC were inversely related to CD133^+^ Fas-L-GSCs. **(H)** T reg PBMCs were inversely related to CD133^+^ c-Met-GSCs except for GSC 29. **(I)** T reg PBMCs were directly related to the CD133^+^ Fas-GSCs. **(J)** CD4^+^ TIL was directly related to the CD4^+^ PBMC. **(K)** CD8^+^ TIL were inversely related to CD8^+^ PBMC. **(L)** T reg-TIL was inversely related to T reg PBMC. **p < 0.01 represent significant difference compared between patients and healthy control.

Similarly, to assess the correlation between the expression of c-Met and FasL on GSCs with peripheral circulating immune cells, PBMCs of the same glioblastoma patients and healthy controls were evaluated for CD4^+^, CD8^+^, and T reg (CD25 + FOXP3) through flow cytometry. Results showed that baseline expression of CD8^+^ (35.24%) and T reg (15.12%) cells was significantly higher in patients with glioblastoma compared to healthy controls (18.05 and 1.67% respectively) (Figure 3A). However, there was no significant difference in the CD4^+^ lymphocyte population.

The CD4^+^ PBMC population presented an inverse relation with the CD133^+^ c-Met GSCs population except for GSC 29 sample. On the contrary, they were directly related to the CD133^+^ FasL GSCs population (Figures 3B,C). The CD8^+^ PBMC population seemed to be inversely related to the CD133^+^ c-Met population except for GSC 29 sample, whereas it was directly related to CD133^+^ FasL GSCs population (Figures 3D,E). Moreover, the CD8^+^ PBMC expressing c-Met and Fas (CD8^+^ ve c-Met PBMC and CD8^+^ ve Fas PBMC) were found to be inversely related with CD133^+^ c-Met and CD133^+^ Fas-L GSCs population (Figures 3F,G). T reg PBMCs were inversely related to the CD133^+^ c-Met GSCs population except for GSC 29 sample; however, they were directly related to the CD133^+^ Fas GSCs (Figures 3H,I).

### Tumor infiltrating lymphocyte and peripheral circulating immune cells in glioma patients are correlated

The trend in the immune cell subsets in TILs and PBMC was evaluated. Results showed that the percent frequency of CD4^+^ TIL was directly related to the CD4^+^ PBMC percent frequency (Figure 3J). On the contrary, the percent frequencies of CD8^+^ TIL and T reg-TIL were inversely related to percent frequencies of peripheral CD8^+^ and peripheral T reg, respectively (Figures 3K,L).

## Discussion

Our findings suggested that the expression of c-Met and FasL on patient-derived GSCs could be associated with the immunomodulation in the tumor microenvironment for glioblastoma patients. The correlation between c-Met and FasL expressing GSCs with frequencies of CD8^+^, CD4^+^, T reg TILs, and microglia confirms the association between GSCs in the immune suppressive phenotype of glioblastoma tumors.

Glioblastoma is one of the most aggressive and infiltrative tumors, and even after surgery and postoperative concomitant radiotherapy and chemotherapy have provided survival benefits, the patient prognosis remains poor (Stupp et al., 2005). Although immunotherapies such as immune checkpoint blockers have shown significant success in several tumors, but have not proven to be very successful in glioblastoma (Antunes et al., 2020). These therapeutic failures can be regarded as the heterogeneous nature of glioblastoma. Several recent studies have explained the role of GSCs in driving the resistance mechanisms in glioblastoma tumors (Ma et al., 2018; Khan et al., 2021). Thus, investigating the TILs in the tumor microenvironment and their association and correlation with tumor cells, especially GSCs, is crucial for designing novel therapeutics. Although few studies have started to emerge that attempted to evaluate the role of TILs in the pathology of glioma tumors, there is no study explaining the association between GSCs and TILs in glioblastoma tumors.

Thus, for the first time, in the present study, we showed that c-Met and FasL antigens expressed on patient-derived GSCs were associated with TIL populations as well as peripheral circulating immune cells. We showed that a large population of GSCs expressed c-Met and FasL antigens. The TILs from glioblastoma tumor specimens showed a high CD4^+^ population accompanied by a low CD8^+^ TIL population, which was consistent with the previous study by Han et al. (2014). The expression of CD4^+^ cells is explained as a double-edged sword in immunological evaluations. On one side, CD4^+^ T cells are crucial for the activation and functioning of CD8^+^ cells. On the other hand, CD4^+^ T reg cells suppress the antitumor immune response (Han et al., 2014). We showed that patients with glioblastoma displayed an immunosuppressive phenotype, both local and systemic immune responses. We observed an inverse relation when we compared the c-Met expressing GSCs population with all CD4^+^ cells and T reg TILs separately.

Moreover, CD8^+^ TIL frequencies were directly related to c-Met expression on GSCs. These findings suggest that c-Met expression on GSCs did not indicate its association with the total CD8^+^ TIL population. As previously indicated, c-Met on tumor cells can act as a tumor-associated antigen (TAA) and elicit a CD8^+^ mediated antitumor response (Kumai et al., 2015). Unlike our results, c-Met is also shown to induce a decrease in interleukin-17 (IL-17) lymphocytes and increased T reg lymphocytes, IL-10 production, and transforming growth factor-beta (TGF-β) along with a reduction in CD8^+^ lymphocyte (Okunishi et al., 2005; Benkhoucha et al., 2010). The CD8^+^ ve c-Met lymphocytes have been shown to produce high levels of interferon-γ (IFN- γ) and granzyme (Benkhoucha et al., 2020). Very recently, Parmigiani et al. have explained the role of IFN-γ in immune suppressive tumor environment in glioma tumors (Parmigiani et al., 2022). Thus, we investigated CD8^+^ ve c-Met TILs correlation with GSCs expressing c-Met, and an inverse relation was found between these populations. These results indicate that c-Met expressing cells may not modulate all CD8^+^ lymphocyte populations but can mediate an immunosuppressive phenotype through modulating the CD8^+^ lymphocyte positive for c-Met expression. Possibly, IFN-γ secreted by CD8^+^ lymphocyte positive for c-Met may mediate the immune suppression mechanism. It is crucial to unravel the molecular mechanism underneath; it may be mediated through the hepatocyte growth factor (HFG) or by the interplay of cytokines in the microenvironment.

It is well-known that several malignant brain tumors, including glioblastoma, present high expression of FasL (Benkhoucha et al., 2020; Parmigiani et al., 2022). In concordance with these studies, we showed that a high population of GSCs expressed FasL. Moreover, our findings showed that FasL expressing GSCs population was inversely related to CD8^+^ lymphocyte frequencies; on the other hand directly correlated with Fas expressing CD8^+^ lymphocytes. It is explained that FasL on tumor cells can induce apoptotic mediated cell death of immune cells through complexing with Fas receptor on immune cells, ultimately leading to immune evasion (Céfai et al., 2001; Didenko et al., 2002). Thus we evaluated CD8^+^ lymphocytes for expression of Fas receptor, and we found that they were directly correlated in three patients suggesting that they mediate the reduction in the cytotoxic CD8^+^ lymphocyte population. Further supporting our hypothesis, FasL expressing GSCs seemed to mediate immune suppression through Fas receptors on CD8^+^ lymphocytes. However, further evaluation is warranted to confirm the mechanism.

Microglia are the main effector immune cell population in the central nervous system (CNS) and constitute a major portion of the TIL population in CNS malignancies (Choi et al., 2001). Badie et al. showed that high expression of FasL in the murine intracerebral tumor was mediated through the microglia population, which further contributes to immunosuppression in the tumor site (Choi and Benveniste, 2004). Moreover, it is known that microglia can induce apoptosis in Fas expressing T lymphocytes (Badie and Schartner, 2000). Concurring with these studies, our findings showed that the expression of FasL GSCs was directly related to microglia TIL populations. We suppose that FasL expressing GSCs could induce the FasL expression in microglia, as previously described by Badie et al. in murine intracerebral tumors, and the FasL expressing microglia mediate apoptosis in CD8^+^ lymphocytes (Badie et al., 1999).

Furthermore, it is reported that c-Met expressing glioma tumors display an increased population of microglia (Badie et al., 2001). Supporting this notion, our findings indicated that c-Met expression on GSCs is directly correlated with the microglia population, suggesting that GSCs expressing c-Met may recruit microglia to the tumor site. However, it would have been better to evaluate the M1 and M2 populations to evaluate the tumor-associated macrophages to get a clearer picture of immunosuppression in the tumor site.

The TIL population in glioblastoma has been documented for their prognostic values in predicting the clinical outcome of patients (Frigerio et al., 2000; Han et al., 2014). Several studies have reported peripheral effector immune cells expressing tumor-associated antigens (Brooks et al., 1978). Here we showed that TIL population, including CD4^+^ and T reg lymphocytes, were directly related to peripheral circulating CD4^+^ and T reg lymphocytes, suggesting their prognostic importance. The CD8^+^ TIL lymphocytes were inversely associated with the peripheral circulating CD8^+^ lymphocytes, possibly due to GSC-mediated reduction in the CD8^+^ cells.

The study has a few limitations, such as; low GSC samples, which limits the study statistically. It can be explained as it is well-known that the chances of GSC neurosphere development from each tumor specimen are meager. We processed a notable number of tumor tissues from 29 patients with glioblastoma, but we only found neurosphere growth in four samples. Secondly, the study limits in a mechanistic aspect of evaluation. It is crucial to understand better the underlying molecular mechanism of FasL and c-Met mediated immunosuppression by GSCs in glioblastoma.

Following the lead from these preliminary finding, it stands out that in future studies fewaspects can be taken in account for; (1) evaluation of the link between CD8^+^ ve c-Met and cytokine, (2) The association between the M1 and M2 macrophages and c-Met and FasL^+^ ve GSCs, and (3) The mechanism of GSC expressed FasL and lymphocyte Fas receptor complex mediated immunosuppression in glioblastoma.

In conclusion, our findings suggested that the expression of c-Met and FasL on patient-derived GSCs could be associated with the immunomodulation in the tumor microenvironment for glioblastoma patients. However, a cohort study with a larger sample size investigating the molecular mechanism is warranted to support these preliminary findings.

## Data availability statement

The original contributions presented in this study are included in the article/supplementary material, further inquiries can be directed to the corresponding author.

## Ethics statement

The studies involving human participants were reviewed and approved by Bezmialem Vakif University Ethics Committee. The patients/participants provided their written informed consent to participate in this study.

## Author contributions

MH: draft revised and edited, study conception, and design. IK, SM, EE, BK, and HK: perform all the experiments. IK: write the first draft of the manuscript. KA and AS: collection of patient samples, patient data, consent forms, and statistical analysis. All authors contributed to the study, read, and approved the present version of the manuscript.
